# 

**Published:** 1890-06-21

**Authors:** 


### NOTICE TO CORRESPONDENTS.

All MS., Letters, Books for Review, and other matters intended for the Editor should be addressed The Editor, The Lodge, Porchester Square, London, W.

The Editor cannot undertake to return rejected M.S., even when accompanied by stamped directed envelope.

**Notice to Advertisers and Subscribers.**

All Advertisements, Orders for Copies of The Hospital, and Business Communications should be addressed to The Publisher, *not to the Editor*, The Hospital, 140, Strand, W.C.

**Bound Volumes of "The Hospital."**

Vol. VI., containing full page portraits of T.R.H. the Prince and Princess of Wales, and Miss Florence Nightingale, is obtainable at 4s., post free.

Orders will be received for Vol. VII., 4s. post free. Cases for binding may be had of the Publisher, at 1s. 6d. each.

**To Residents, Secretaries, and Matrons.**

The Editor will be glad to have the Regulations and each Report as issued by Asylums, Hospitals, Convalescent and Nursing Institutions, as well as those of other Charities, and an early copy in duplicate of all Appeals and Festival Notices, etc., addressed to *The Editor, The Lodge, Porchester Square, London, W.* Any superintendent, secretary, matron, or other chief official who regularly sends such information may be registered, on application to the Publisher, to receive free of cost a cover for binding The Hospital in half-yearly volumes.

### BURDETT'S HOSPITAL ANNUAL FOR 1890

Is now ready, and may be obtained of the Publisher, The Hospital Offices, 140, Strand, W.C., price 2s. 6d. limp boards, or 3s. 6d. cloth. All orders must be accompanied by a remittance.

### BOOKS RECEIVED.

We have recently received the following books for review:

Alexander and Shepheard.

"The Cure of the Skin in Health and Disease." F. Augustus Cox, M.B.

Authors' Co-Operative Publishing Co. (Limited).

"A City Girl." John Law.

Baillière, Tindall, and Co.

"Detection of Colour Blindness." F. W. Edridge-Green.

Cassell and Co.

"Metropolitan Year Book for 1890."

"Christian Commonwealth" Publishing Co. (Limited).

"Faith Cures: Their History and Mystery." A. J. L. Gliddon.

Churchill, J. and A.

"Guy's Hospital Report, 1889."

Clarendon Press.

"Wiseman on Heredity." Translated by Poulton, Schönland, and Shipley.

Gill, L. Upcott.

"House Medicine and Surgery." W. J. Mackenzie.

"Mediterranean Winter Resorts." E. A. Reynolds Ball.

Kegan Paul, Trench, and Co.

"Physiology of Bodily Exercise." Fernand Lagrange.

Kenning, Geo.

"Exercises in Practical Chemistry." Dr. W. K. Hodgkinson.

Longmans, Green, and Co.

"A Manual of Anatomy." Edmund Owen, M.B., F.R.C.S.

"National Health." B. Ward Richardson, M.D., F.R.S.

"School Hygiene." W. J. Abel.

Macmillan and Co.

"Captain Cook." Walter Besant.

"From Strength to Strength." J. B. D.

"Middle-Class Cookery Book."

"Text Book of Pathology." Vol. I. Hamilton.

Nelson and Sons.

"Bible Illustrations from the New Hebrides." Rev. J. Inglis, D.D.

Open Court Publishing Co.

"Psychology of Attention." Ribot.

P. and O. Pocket Book.

Philip, George, and Son.

"Educational Annual for 1890."

Relfe Bros.

"Modern Thoughts and Modern Thinkers." J. F. Charles.

Remington and Co.
"A Fairy Godmother." J. A. Goodchild.
Scott, Walter.
"Physiognomy and Expression." P. Mantegazza.
Spon, E. F. and N.
"Bayley's Pocket Book for Pharmacists," &c. Thos. Bayley.
"Health and Comfort in House Building." Drysdale and Hayward.
Stock, Elliot.
"Abolition of Suffering." S. B. G. M'Kinney, M.A.
Stott, David.
"Food in Motherhood." Ephraim Cutler.
Swan Sonnenschein and Co.
"Browning's Message to his Time." Edward Berdoe.
"Habit and Health." Guy Beddoes.
"The Healing Art and the Claims of Vivisection." Edward Berdoe.

### The Hospital Workshop.

### Scraps and Gleanings.

Hospital Saturday will be kept at Croydon on July 5th.

The Hospital Sunday Fund had, on Wednesday, nearly reached £30,000.

Oswestry Provident Dispensary has now £1,000 invested, and £240 in hand.

Lewisham Guardians have adopted the scheme for the new workhouse infirmary.

Dr. Thorne Thorne and Dr. David Sharp have been elected Fellows of the Royal Society.

Mrs. Yerborough has bequeathed two annuities of £100 and £500 to Blackburn Infirmary.

Kettering is again in a state of agitation about a proposal to establish a cottage hospital.

The late Miss Jane Clarke, and Mrs. Mary Curtis have left large bequests to medical charities.

At Aston 234 persons have been examined by the St. John Ambulance Association since November, 1887.

The Duke of Bavaria is a celebrated oculist; while staying in the Tyrol lately he performed 253 operations on the eye, free.

The Earl of Rosebery laid the foundation-stone of the first lunatic asylum which the new County of London is erecting for pauper lunatics.

Surgeon-Major W. F. Stevenson has been appointed Assistant-Professor of Clinical and Military Medicine at the Army Medical School, Netley.

The annual meeting of the Hospital Saturday Committee was held at Leamington lately, Dr. Thursfield presiding. The report was satisfactory.

The Royal Dispensary, now a purely benevolent institution, is likely to become a provident institution. This is a move in the right direction.

The annual dinner of the Irish Medical Schools and Graduates Association was held lately at Bath. Several ladies were present, including Dr. Eliza Walker.

During the absence of her nurse, Miss Mary McGaw, a well-known Manchester philanthropist, who had lately suffered from melancholia, committed suicide.

Baron Ferdinand de Rothschild presided at the dinner in aid of the City Orthopædic Hospital, when an appeal for £10,000 for building purposes was made.

Hospital Saturday was held at Sunderland last week. Two Norwegian sailors, who could speak all languages, made a special collection in the docks.

Chiseldon has held its first Hospital Saturday; a procession, a public tea, and a concert resulted in a collection of £22 for the benefit of the Swindon and Savernake Hospital.

On the 10th inst. a garden party was given at Walton on-Thames in aid of the Metropolitan Convalescent Institution, and was graced by the presence of the pretty Duchess of Albany.

Towend Cottage Hospital, Dumbarton, was open for inspection last week. It is a picturesque little building, erected from designs by Messrs. Shiells and Thomson, of Edinburgh.

The Hospital for Epilepsy and Paralysis issues its report in quaint parchment covers. It is an excellent tale of work well done; the in-patients numbered 115, and the out-patients 9,000.

Surgeon Parke was entertained on Monday at dinner in the Shelbourne Hotel, Dublin, by the officers of the Army Medical Corps. Covers were laid for eighty, and Surgeon-General Lindsay presided.

Mr. and Mrs. Oakes, of Holloway, who, as alleged, poisoned their son and attempted self-destruction both by poison and by cutting their throats, are recovering from their injuries under the care of the medical staff at the Great Northern Central Hospital.

It is stated that by the death of Mrs. Hopper, widow of the Rev. R. L. Hopper, formerly vicar of St. George's, Brandon-hill, Bristol, the Irish Church Missionary Society will benefit to the extent of £40,000, and that handsome legacies are also left to the Bristol General Hospital and Royal Infirmary.

The arrangements for the fifty-eighth annual meeting of the British Medical Association (under the presidency of Dr. W. F. Wade, senior physician to the Birmingham General Hospital), to be held in Birmingham on July 29th, 30th, and 31st, and August 1st next, are making satisfactory progress.

Petitions signed by members of the medical profession have been presented to the House of Lords, praying that the inquiry of the Select Committee regarding Metropolitan Hospitals, &c., may be extended so as to embrace the provincial medical charities. The petitions have been referred to the Committee.

The German dentist, Hermann Arnemann, who was sentenced recently to twenty years' penal servitude for shooting Judge Bristowe at Nottingham, was found dead in his cell at Leicester Gaol, having hanged himself to the ventilator by means of his braces. He had served nearly nine months of his sentence, and had appeared cheerful.

The Duke of Devonshire has lent Devonshire House, Piccadilly, on the afternoon of Thursday, June 26th, for a meeting in aid of the Nurses' Convalescent Home. The committee has Princess Christian for its president, and the Duchess of Abercorn, Lady Edward Cavendish, Lady Audrey Buller, and Lady George Hamilton among its members.

The Prince of Wales will lay the foundation-stone of the new buildings for the Royal South London Ophthalmic Hospital in July, when the Princess will receive purses of £5 5s. and upwards from ladies and children, who will present them to her on behalf of the Royal Foundation Fund. The purses will be returned to the collectors after the ceremonial, with inscriptions as mementoes of the day.

